# Posterior and anterior tilt increases the risk of failure after internal fixation of Garden I and II femoral neck fracture

**DOI:** 10.1080/17453674.2019.1637469

**Published:** 2019-07-04

**Authors:** Pontus Sjöholm, Volker Otten, Olof Wolf, Max Gordon, Gustav Karsten, Olof Sköldenberg, Sebastian Mukka

**Affiliations:** a Department of Surgical and Perioperative Sciences at Umeå University, Umeå;; b Section of Orthopaedics, Department of Surgical Sciences, Uppsala University, Uppsala;; c Departments of Orthopedics and Clinical Sciences at Danderyd Hospital, Karolinska Institute, Stockholm, Sweden

## Abstract

Background and purpose — Preoperative posterior tilt of the femoral head as seen on lateral radiographs has been reported to affect the risk of fixation failure in cases of minimally displaced femoral neck fractures (Garden I–II). We investigated radiological risk factors of treatment failure.

Patients and methods — We included 417 patients (68% women, median age: 78 years (50–108) with a minimally displaced femoral neck fracture (Garden I–II) treated with internal fixation in a retrospective cohort study. The patients were followed for 3.4 years (2–14). Data on age, sex, housing, cognitive impairment, implant angulation, pre- and postoperative tilt, hip complications, and reoperations were recorded. The risk of fixation failure was assessed using Cox proportional hazards regression analysis.

Results — The overall reoperation rate was 17%, and the rate of treatment failure (fixation failure, nonunion, avascular necrosis, or posttraumatic osteoarthritis) was 13%. Cox proportional hazard analysis revealed an increased risk of treatment failure with a preoperative posterior tilt of at least 20° and a preoperative anterior tilt greater than 10°. A failure occurred in 13 of the 65 patients with a posterior tilt of at least 20° and in 5 of the 9 patients with an anterior tilt greater than 10°.

Interpretation — A preoperative posterior tilt of 20° and an anterior tilt greater than 10° in cases of Garden I and II femoral neck fractures increase the risk of fixation failure necessitating additional surgery. In this group of patients, there is a need for future interventional studies regarding the feasibility of primary hip arthroplasty.

Several authors have raised doubts regarding the results of the internal fixation of minimally displaced femoral neck fractures (FNFs) (Rogmark et al. 2009, Gjertsen et al. 2011). In elderly patients, reoperation rates ranging from 8% to 19% have been reported (Onativia et al. 2018). Several authors have proposed that preoperative posterior tilt of the femoral head increases the risk of reoperation after internal fixation of minimally displaced FNFs (Palm et al. 2009, Clement et al. 2013, Dolatowski et al. 2016). However, Lapidus et al. (2013) were unable to reproduce these findings. Surgeons need a reliable predictor that can be used to identify patients who are at risk of treatment failure after internal fixation.

We further investigated the value of posterior or anterior tilt and comorbidities for predicting fixation failure and avascular necrosis (AVN) after internal fixation of minimally displaced FNFs. We hypothesized that the risk of failure would increase with increasing preoperative tilt of the fracture.

## Patients and methods

### Study design and setting

This retrospective cohort study included patients treated with closed reduction and internal fixation of minimally displaced FNFs between 2003 and 2015 at the Orthopaedic Department of Umeå University Hospital, Sweden. Umeå University Hospital is a third-level university hospital with a catchment area of emergency care for approximately 160,000 inhabitants. The STROBE guidelines were followed.

### Participants and data collection

We included all patients with an age greater than 50 years and acute minimally displaced FNF (Garden I–II) treated with internal fixation between 2003 and 2015 in our department. Patients were followed until February 2019 or until death. Data were collected retrospectively throughout the study period by a combination of in-hospital surgical and medical records at admission. Patient data included age, sex, cognitive impairment (yes/no, classified by the treating surgeon; temporary confusion was not classified as cognitive impairment), use of a walking aid, sheltered living (yes/no), surgical treatment, and date of death.

### Radiographic analysis

The anteroposterior (AP) radiographs were used to classify fractures according to the Garden system as minimally displaced (Garden I–II) or displaced (Garden III–IV) (Nilsson et al. 1993). The preoperative tilt angle of the femoral head was measured on a lateral radiograph of the hip using the method described by Palm et al. (2009). Implant positioning on the postoperative AP pelvic radiograph was divided into 2 categories (inclination of ≤ 125° and > 125°).

All images were digitally acquired using the Picture Archiving and Communication System (PACS, Impax, Agfa, Antwerp, Belgium), and all measurements were performed by PS who was not blinded to the outcome.

### Implants and surgery

Internal fixation was carried out with the patient on a fracture table. Before sterile draping, the fracture underwent closed reduction if angulated and amenable to closed reduction with the aid of an image intensifier. Internal fixation was performed with 2 pins (Hansson Pins, Swemac, Sweden). On the AP projection, the caudal pin was aimed to extend from the level of the lesser trochanter laterally along the medial inferior cortex of the femoral neck to approximately 1 cm from the border between the bone and cartilage of the head. The cranial pin was positioned parallel at 1 to 2 cm dorsoproximal from the distal pin. The pins were parallel and positioned in the central or posterior third of the femoral head and neck. Neither capsulotomy nor joint aspiration was performed.

Antibiotic prophylaxis with cloxacillin was given on the day of surgery. Low-molecular-weight heparin was administered postoperatively for 14 days. The aim was to mobilize all patients on the first postoperative day under the supervision of a physiotherapist.

### Outcomes

Treatment failure was defined as fixation failure, nonunion, avascular necrosis (AVN), or posttraumatic osteoarthritis. Major reoperation was defined as hip replacement or excision arthroplasty. Minor reoperation was defined as pin removal. Complications were defined as all registered complications related to the hip, i.e., fixation failure, nonunion, AVN, posttraumatic osteoarthritis, and peri-implant fracture.

### Statistics

We used a Cox proportional hazards regression model with time since primary surgery as the dependent variable with time to whichever of the following events first occurred: reoperation, death, or uneventful end of the observational time. We evaluated the proportional hazards assumption using Schoenfeld residuals.

In addition to the exposure variable (posterior and anterior tilt) we selected age, sex, sheltered housing or nursing home, and cognitive impairment as covariates for fragility. As surgical factors may impact the risk for failure, we also included the inclination angle (Nyholm et al. 2018) and postoperative tilt of the pins to adjust for surgical factors that might affect the risk of failure. We performed an unadjusted relative risk calculation.

Pre- and postoperative tilt, implant angulation, and age were modeled as continuous variables where each was tested for non-linearity using ANOVA, and, if significant, they were modeled using restricted cubic splines, where the number of knots, which determines flexibility of the spline, was chosen using the Bayesian information criterion (BIC), also known as Schwarz’s information criterion. We used R version 3.5.2 in combination with the rms package (v. 5.1–2; R Foundation for Statistical Computing, Vienna, Austria) for the survival analysis.

### Ethics, data sharing, funding, and potential conflicts of interest

The study was conducted in accordance with the ethical principles of the Helsinki Declaration and was approved by the Ethics Committee of Umeå University (entry number dnr 2017-489-32M and 2016-447-31M). Data will be made available on request to the corresponding author. The study was funded by grants from the regional agreement on medical training and clinical research (ALF) between Västerbotten County Council and Umeå University. The authors declare no competing interests.

## Results

### Patients and descriptive data

**Figure 1.**
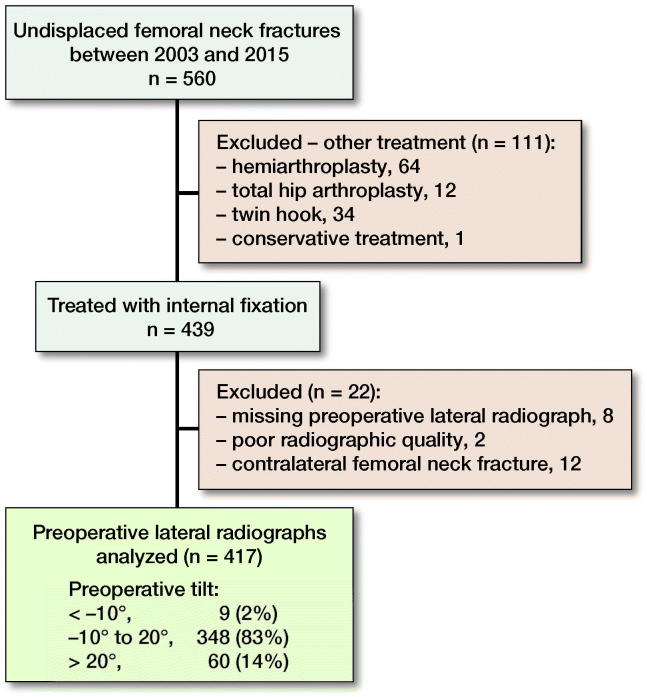
Flowchart of patient selection.

417 patients (median age: 78 years [50–108] with a mean follow-up of 3.4 years [2–14]), 68% of whom were women, were included in the study (Figure 1, Table 1). The mortality rate was high, with an overall 1-year mortality rate of 20% and a 2-year mortality rate of 33%.

**Table 1.** Characteristics of patients (n = 417)

Age^a^	78 (10)
Women^b^	285 (68)
Cognitive impairment^b^	165 (40)
Sheltered housing^b^	153 (37)
Preoperative tilt^a^	10° (10)
Missing^b^	0
Postoperative tilt^a^	5° (8)
Missing^b^	5 (4)
Implant angulation^a^	141° (7)
≤ 125°^b^	7 (2)
> 125°^b^	399 (96)
Missing^b^	11 (3)

a Mean (SD).

b n (%).

### Complications

The overall complication rate during the study period was 14.5%, including 4% fixation failure, 2% nonunion, 6% AVN, 0.5% posttraumatic osteoarthritis, and 2% peri-implant fracture. All fixation failures occurred during the first year, all cases of AVN and the 2 cases of posttraumatic osteoarthritis occurred during the second year or later.

### Reoperations

**Figure 2.**
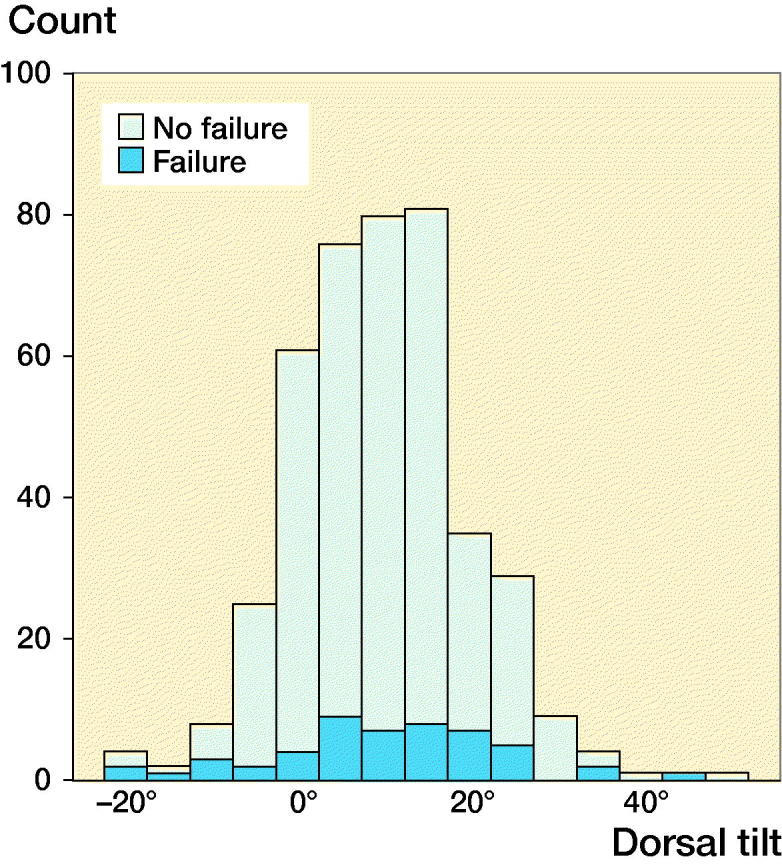
Diagram displaying the distribution of patients and treatment failure (n = 417).

17% of all patients underwent reoperation, including minor procedures, such as screw removal, during the study period (Figure 2, Table 2). 2 patients with nonunion and 2 patients with AVN did not undergo surgery due to their general health status (Table 2).

**Table 2.** Treatment failures and reoperations

	< –10°(n = 9)	–10° to 20°(n = 348)	> 20° (n = 60)
Age^a^	80 (4)	78 (10)	78 (10)
Women^b^	8 (89)	242 (70)	35 (58)
Failure^b^	5 (56)	35 (10)	13 (22)
Type of failure, n			
Fixation failure	2	8	6
Nonunion	1	7	2
Avascular necrosis (AVN)	2	18	5
Posttraumatic osteoarthritis	0	2	0
Reoperation^b^	5 (56)	49 (14)	15 (25)
Type of reoperation, n			
Removal of osteosynthesis	0	16	3
Hemiarthroplasty	3	6	4
Total hip arthroplasty	2	17	7
Revision osteosynthesis	0	2	0
Peri-implant fracture	0	6	1
Girdlestone	0	2	0

a Mean (SD).

b n (%).

### Risk of treatment failure

**Figure 3.**
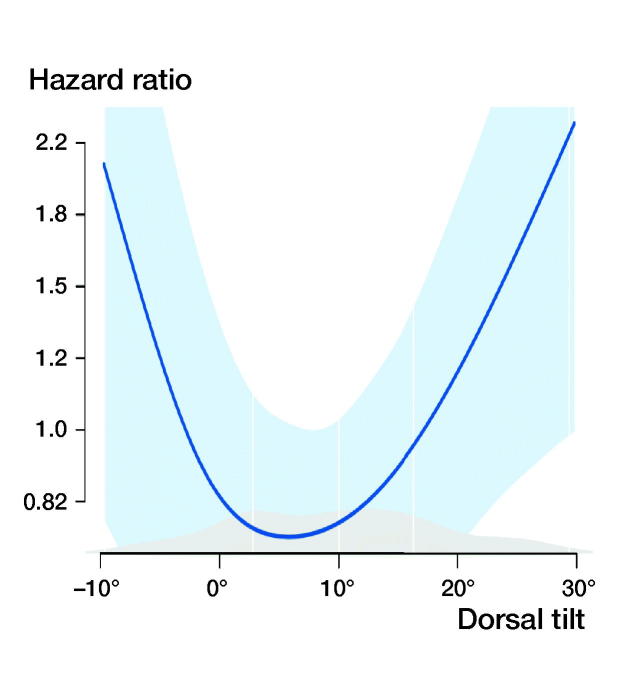
Plot of the risk of treatment failure. Anterior tilt corresponds to negative values on the X-axis. Adjusted for sex, sheltered housing, cognitive impairment, implant angulation, and postoperative tilt. Light blue area corresponds to 95% CI.

13% of patients underwent major reoperation and/or were diagnosed with fixation failure, nonunion, AVN, or posttraumatic osteoarthritis. The relative risk (RR) of treatment failure for patients with a preoperative tilt of more than 20° was 2.2 (95% CI 1.2–3.8) and the RR for an anterior tilt of greater than –10° was 5.5 (CI 2.8–11) compared with patients with a preoperative tilt of –10° to 20° (Figure 3, Table 3).

**Table 3.** Cox proportional hazard model for covariates associated with treatment failure adjusted for sex, sheltered housing, cognitive impairment, implant angulation, and postoperative tilt

Variable	Crude	Adjusted
	HR (2.5%–97.5%)	HR (2.5%–97.5%)
Age	1.0 (1.0–1.0)	1.0 (1.0–1.1)
Sex		
Female	1.0 Ref.	1.0 Ref.
Male	0.7 (0.3–1.3)	0.6 (0.3–1.3)
Sheltered housing:		
No	1.0 Ref.	1.0 Ref.
Yes	0.5 (0.2–1.0)	0.4 (0.2–1.1)
Cognitive impairment:		
No	1.0 Ref.	1.0 Ref.
Yes	0.7 (0.4–1.4)	0.9 (0.4–2.0)
Implant angulation	1.0 (1.0–1.0	1.0 (1.0–1.0)
Preoperative tilt:		
–20°	5.8 (1.7–19)	7.9 (1.8–35)
–10°	2.9 (1.4–6.3)	3.6 (1.4–9.3)
0°	1.5 (1.1–2.1)	1.6 (1.1–2.5)
10°	1.0 Ref.	1.0 Ref.
20°	1.5 (1.2–2.0)	1.5 (1.1–2.1)
30°	3.3 (1.7–6.4)	3.4 (1.6–7.3)
40°	7.0 (2.4–21)	7.5 (2.2–25)
Postoperative tilt	1.0 (1.0–1.0)	1.0 (1.0–1.1)

HR = hazard ratio; n = 417.

## Discussion

In this retrospective cohort study, we found that the risk of treatment failure was higher for the one-fifth of patients with a preoperative anterior tilt of at least 10° or posterior tilt of at least 20°. We suggest that a thorough preoperative assessment of the lateral radiograph be performed in cases of a minimally displaced FNF in elderly patients to define who should be considered for arthroplasty rather than internal fixation.

The impact of posterior tilt has been debated previously, and studies have presented various results (Palm et al. 2009, Clement et al. 2013, Lapidus et al. 2013, Dolatowski et al. 2016). The differences among the conclusions might be attributed to differences in the categorization of measurements, definition of outcomes and reoperation, number of included patients, and follow-up durations. Among patients with a posterior tilt exceeding 20°, Palm et al. (2009), Lapidus et al. (2013), and Dolatowski et al. (2016) reported a reoperation rate of 56%, 10%, and 19%, respectively. In concordance with the report by Dolatowski et al., we found a similar failure rate. Palm et al. (2009) included 3 peri-implant fractures as failures; in contrast, Lapidus et al. (2013) analyzed reoperation rates and excluded 5 patients for whom revision surgery was indicated but not performed due to medical comorbidities. Clement et al. (2013) included screw removal due to local discomfort, thus increasing the failure rate.

An anterior tilt of at least 10° has not been described and published in the peer-reviewed literature as a risk factor of treatment failure, although this phenomenon has been proposed in a book discussing internal fixation for FNFs by Manninger and coauthors (2007). We found, somewhat surprisingly, that a posterior tilt up to 10 degrees gave the lowest risk for failure. This might be due to an increase in compression and stability at the fracture site.

Fracture reduction did not decrease the risk of treatment failure, which is in agreement with the results previously reported (Palm et al. 2009, Dolatowski et al. 2016). A more pronounced tilt might cause a predisposition to greater instability due to comminution of calcar femorale and thus lead to an increased risk of disrupted healing tendency for redisplacement and mechanical failure (Alho et al. 1992).

In a recent study by Nyholm et al. (2018), the risk factors of reoperation were analyzed in patients undergoing osteosynthesis for a Garden I–II or Garden III–IV FNF with parallel implants. The authors concluded that insufficient reduction, varus implant positions (≤ 125°), and femoral head cartilage perforation were the only surgical factors influencing the risk of reoperation. Thus, implant placement in minimally displaced femoral neck fractures might be of less importance than previously suggested. In our study, we found no statistically significant correlation between the risk of reoperation and postoperative tilt or varus implant positions.

Hip arthroplasty is the treatment of choice for elderly patients with displaced FNFs (Rogmark and Leonardsson 2016). In elderly patients, failed internal fixation necessitating a revision hip arthroplasty is a severe complication. Also, salvage arthroplasty following failed internal fixation has inferior outcomes compared with primary hip arthroplasty (Blomfeldt et al. 2006, Frihagen et al. 2007). In groups with a high risk of fixation failure, such as patients with an anteriorly or posteriorly tilted FNF (Garden I–II), hip arthroplasty is a feasible option that could improve the surgical outcome.

In a recently published randomized controlled trial comparing hemiarthroplasty with screw fixation for Garden I–II FNFs, the authors found similar hip function, as measured by the Harris hip score (Dolatowski et al. 2019). However, regarding secondary outcomes, hemiarthroplasty led to improved mobility and fewer major reoperations. The authors also performed a post-hoc analysis of those patients allocated to internal fixation and found that those with a preoperative posterior tilt of more than 20° had a higher risk of healing-related complications compared with those with a preoperative tilt of 20° or less. Further large register-based randomized controlled trials focusing on high-risk groups are of interest for establishing whether the reduced reoperation rate and improved mobility lead to improved patient-reported outcomes.

Inherent flaws of the retrospective observational study design and the lack of regular or scheduled follow-ups limit the impact of our study. There are failures that were not identified due to the lack of scheduled follow-ups and would consequently increase the failure rate. We found a tendency towards the risk of treatment failure being lower for patients living in sheltered housing, most likely explained by the high mortality rate and the fragility of patients unfit to actively seek healthcare services. This reduces the identification of failure and masks the breadth of issues related to the internal fixation of minimally displaced FNFs. All measurements were performed by 1 rater. We did not perform any validation of the measurements; however, the inter- and intra-rater reliability of posterior tilt measurements in cases of minimally displaced FNFs has previously been reported to be excellent although the repeatability and agreement by the minimal detectable change were found to be 14° (Palm et al. 2009, Dolatowski and Hoelsbrekken 2017).

In summary, patients with a minimally displaced FNF (Garden I–II) with a preoperative posterior tilt of more than 20° or an anterior tilt greater than 10° have an increased risk of fixation failure necessitating a salvage procedure. Primary hip arthroplasty is a feasible option for this group of patients.

The authors thank Olle Svensson at the Section of Orthopedics, Department of Surgical and Perioperative Sciences at Umeå University, for his work with Umanhip.

PS collected and analyzed data, and wrote the manuscript. VO, OW, and OS wrote the manuscript. MG performed the statistical analysis and wrote the manuscript. GK collected data and reviewed the manuscript. SM initiated and supervised the study, collected data, performed the statistical analysis, and wrote the manuscript.

*Acta* thanks Filip C Dolatowski, Frede Frihagen and Jan-Erik Gjertsen for help with peer review of this study.
